# Effect of rearing conditions on the correlation between larval development time and pupal weight of the rice stem borer, *Chilo suppressalis*

**DOI:** 10.1002/ece3.4697

**Published:** 2018-11-20

**Authors:** Xiao‐Long Huang, Lan Xiao, Hai‐Min He, Fang‐Sen Xue

**Affiliations:** ^1^ Institute of Entomology Jiangxi Agricultural University Nanchang China; ^2^ Foreign Language School Jiangxi Agricultural University Nanchang China

**Keywords:** *Chilo suppressalis*, feeding method, larval development time, pupal weight, temperature

## Abstract

A strong positive correlation between development time and body size is commonly assumed. However, the evidence is increasing that the correlation between the two traits can be positive, zero or negative, depending on whether the two traits are under antagonistic or synergistic selection. In the present study, we examined the relation between larval development time and pupal weight of the rice stem borer *Chilo suppressalis* under laboratory and field conditions. For individuals reared at constant temperatures (22, 25, 28 and 31°C), a longer larval period tended to result in larger pupae, showing a positive correlation between larval development time and pupal weight; whereas for those reared under field conditions, a longer larval period tended to result in smaller pupae at 23.5 and 29.8°C, showing a negative correlation between the two traits. There was no correlation between the two traits at the mean daily temperature of 31°C. At constant temperatures, larval development time shortened significantly as rearing temperature increased, whereas pupae tended to become larger at higher temperatures, although no significant difference was detected among temperatures for pupal weight. Under field conditions, larval development time decreased significantly as the mean daily temperature increased, whereas pupal weight of females increased significantly with the increase in the mean daily temperature, which is an example of the reverse temperature–size rule. Feeding method significantly affected larval development time and pupal weight. For individuals fed on live rice plants, larval development time shortened significantly and pupal weight increased significantly compared with those reared on fresh rice stems.

## 1 INTRODUCTION

Development time and body size are two of the most important correlated life history traits and both have profound effects on organism fitness (Abrams, Leimar, Nylin, & Wiklund, 1996; Nylin & Gotthard, 1998; Roff, 2000). Because body size affects reproductive capacity (Davidowitz, 2008; Honek, 1993; Kingsolver & Pfennig, 2004) and development time is related to generation time (Roff, 1992), both can affect the rate of population increase or the rate of spread of favorable genes. Increase in body size and decrease in development time are the usual result of natural selection acting on individual organisms (Eck, Shaw, Geyer, & Kingsolver, 2015; Harrison, Cease, VandenBrooks, Albert, & Davidowitz, 2013; Kingsolver & Huey, 2008; Kingsolver & Pfennig, 2004).

The temperature–size rule in animals describes a common pattern of phenotypic plasticity in which an increase in temperature, within a favorable range, increases metabolic rate and as a consequence, results in higher growth rate, shorter development time, and smaller body size (Sibly & Atkinson, 1994). Such a pattern of phenotypic plasticity has been observed in 75% of insects species studied (Atkinson, 1994). A reasonable assumption is that the relation between development time and body size should be positive, when other conditions remain the same; thus, animals with a longer period of growth should be larger (Nijhout, Roff, & Davidowitz, 2010) or “for one must grow for a longer time to get larger” (Roff, 1992; Stearns, 1992). This assumption may be correct when the relationship between development time and body size within populations is compared along a temperature gradient. However, individual organisms within one population may vary from one another in traits under the same conditions, including development time (Danks, 1987). Development time often varies greatly among individuals, particularly at lower temperatures in some lepidopteran species (Danks, 1987). For example, a difference of 16–19 days was observed in larval development time (the period of larval growth from hatching to pupation) for the rice stem borer *Chilo suppressalis* (Shen & Xue, 1988). Then, do those individuals with longer development times eventually grow and become larger than those with shorter development times? In some insect species, the answer to this question is no. In the Asian corn borer *Ostrinia furnacalis*, four populations from tropical to temperate regions all showed a negative correlation between development time and body weight when larvae were reared under L16:D8 at 26°C, i.e., body weight tended to decrease with increases in development time (Xiao, Fu, He, & Xue, 2014). Such a negative correlation between development time and body size has been found in several insect species, which include the milkweed bug *Oncopeltus fasciatus* (Dingle et al., 1988), the southern green stink bug *Nezara viridula* (McLain, 1991), the pomace fly *Drosophila melanogaster* (Gebhardt & Stearns, 1993), the water strider *Gerris buenoi* (Klingenberg & Spence, 1997), the flush‐feeding geometrid *Epirrita autumnata* (Kause, Saloniemi, Haukioja, & Hanhimäki, 1999), the birch‐feeding sawfly *Priophorus pallipes* (Kause & Morin, 2001), and the scorpion fly *Panorpa cognate* (Engqvist, 2007).

Several studies also show that nutrient quantity and quality of food for insects can change the correlation between development time and body size. Using six sawfly species, Kause et al. (2001) tested the influence of seasonal variation in foliage quality of birch on the relationship between larval development time and body size. When the larvae fed on mature birch leaves of stable quality, strong positive correlations were observed between larval development time and body size. However, when the six species fed on growing or senescent leaves, the correlations between the two traits were negative or zero. Similar results are reported for the yellow dung fly *Scatophaga stercoraria* (Blanckenhorn, 1998) and the cellar spider *Pholcus phalangioides* (Uhl, Schmitt, Schäfer, & Blanckenhorn, 2004) in which the correlations between development time and body size were positive only at low food availability and were weak when food was plentiful. In *Drosophila mercatorum*, the correlation between development time and weight changed from significantly positive when fed high levels of yeast to significantly negative when fed low levels of yeast (Gebhardt & Stearns, 1988). In the butterfly *Pararge aegeria*, development time and pupal weight were not correlated (Gotthard, Nylin, & Wiklund, 1994). More recently, a literature‐derived database of body sizes and development times for over 200 insect species showed that negative correlations between size and development time in response to variations in diet quality are ubiquitous (Teder, Vellau, & Tammaru, 2014).

The rice stem borer, *C. suppressalis* (Walker; Lepidoptera: Pyralidae), is one of the most economically important pests of rice plants in China, with a broad geographic range from southern to northern China (Fu, He, Zhou, Xiao, & Xue, 2016; Xiao, Mou, Zhu, & Xue, 2010). This borer undergoes facultative diapause as full‐grown larvae in response to short day lengths during the autumn (Shen & Xue, 1988; Xiao et al., 2010). In the present study, we investigated larval development time and pupal weight of a subtropical population of *C. suppressalis* originated from the suburbs of Nanchang City (28°46′N, 115°59′E) at different constant temperatures in the laboratory and under field conditions. This population exhibits mixed voltinism, varying from two to four generations per year because of differences in diapause intensity of overwintering larvae and individual differences during larval development (Shen & Xue, 1988; Xiao et al., 2010). Therefore, this population experiences a broad range of temperatures in the field. Studying individual differences in the performance of the two traits may help analyze the influence of a warmer global climate on the relationship between larval development and body size.

## 2 MATERIALS AND METHODS

### 2.1 Experimental insects

Adults used in this study were collected from light traps in late June (the second generation), late July (the third generation) and early September (the fourth generation) 2016 at the rice field of Jiangxi Agricultural University (Nanchang, China). Adults were placed individually into plastic petri dishes (height: 1.5 cm; diameter: 9.0 cm) lined with filter paper and fresh rice leaves to produce egg masses under natural conditions. Egg masses laid on the first 2 days were collected for use in the experiments. The rice variety used in all experiments was mid‐season hybrid rice (Jing4155S×HuaZhan).

### 2.2 Larval development time and pupal weight at constant temperatures

Heads of larvae were black immediately before hatching on July 5, and these egg masses were inserted into leaf sheaths of rice stems with a root collected from the field (here called fresh rice stems) to hatch. The rice stems were transferred to glass bottles (diameter: 12 cm; height: 22 cm) with a thin sheet of water covering the bottom of the bottle. The glass bottles were placed in illuminated incubators (LRH‐250‐GS; Guangdong Medical Appliances Plant, Guangdong, China) under a diapause‐averting photoperiod of L:D 16:8 hr at 22, 25, 28 and 31°C. When larvae developed into third larval instars, they were transferred to plastic boxes (diameter: 16 cm; height: 10 cm) with fresh rice stems for pupation. Each box contained 30 individuals. Each treatment was replicated three to five times. The boxes were checked daily and supplied with new rice stems as required. Upon pupation, the time was recorded, and new pupae were placed individually in cell culture plates with 12 holes (for each hole: diameter: 2.4 cm; height: 2 cm). Pupae were weighed on the 2nd day after pupation using an electronic balance (AUY120; Shimadzu, Tokyo, Japan).

### 2.3 Larval development time and pupal weight under field conditions

To examine the effects of seasonal variations on the relationship between larval development time and pupal weight, the development time and weight for three generations were determined under field conditions. Egg masses immediately before hatching on July 5 (the second generation), August 5 (the third generation) and September 8 (the fourth generation) were inserted into rice leaf sheaths (here called live rice plants) that were cultured in rice pools (for each pool: 1 m^2^ area covered with a net 1 m in height). At least four rice pools were used for each generation. The fourth generation larvae hatched on September 8 are destined to enter diapause when they experience the gradually shortened autumn daylengths in the field (Xiao et al., 2010). To ensure the larvae hatched on September 8 developed to pupation without diapause, the day length for larval growth was prolonged 1.5 hr using 20 W fluorescent lamps. Time to pupation was recorded individually for each larva. Pupae were weighed on the 2nd day after pupation. The data of the mean daily temperature experienced by larvae for each generation were collected from the weather station of Jiangxi Agricultural University.

Furthermore, we compared the differences in development time and body weight for the larvae that hatched on August 5 reared with live rice plants or fresh rice stems.

### 2.4 Statistical analyses

Statistical analyses were conducted using the SPSS 17.0 statistical software package (IBM, www.ibm.com). The correlations between development time and body weight were calculated using linear regressive analysis. The Kolmogorov–Smirnov tests were utilized for the normality distribution confirmation. Bartlett's test and Variance Ratio test were used for the confirmation of equal variances before one‐way analysis of variance (ANOVA). When the hypothesis of normality or equal variances was rejected, the Wilcoxon Mann‐Whitney test was applied to compare the differences in development time and body weight among temperatures at the 5% level of significance.

## 3 RESULTS

### 3.1 Larval development time and pupal weight at constant temperatures

**Figure 1:**
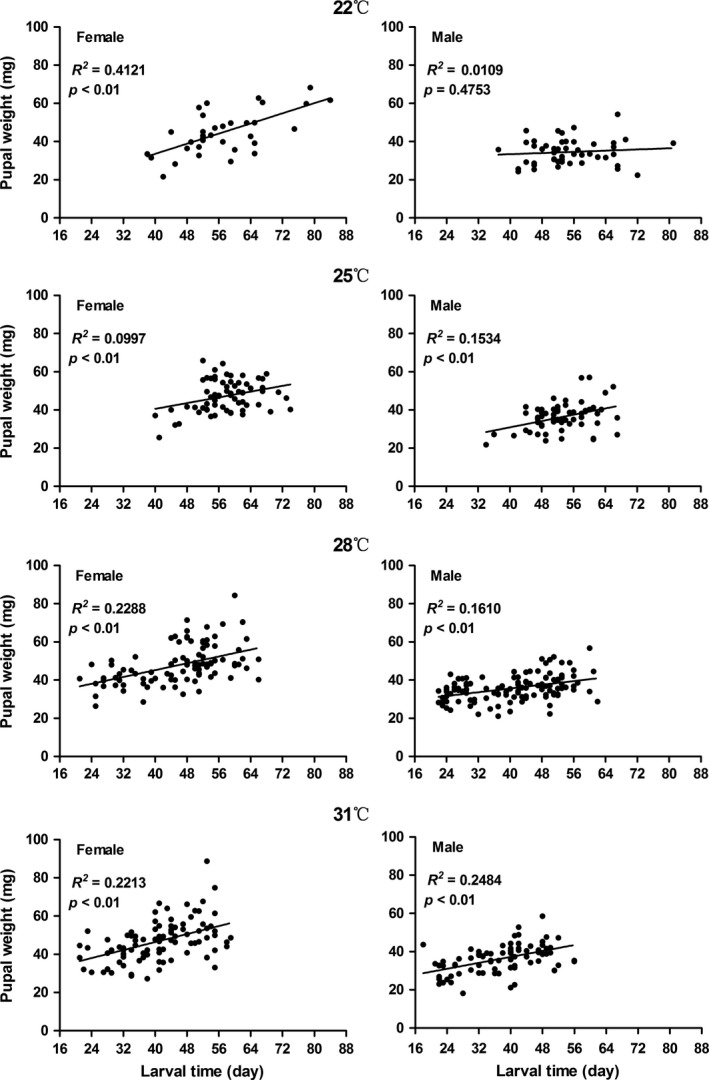
Correlation between larval development time and pupal weight of the rice stem borer *Chilo suppressalis* at constant temperatures

As shown in Figure 1, the correlations between larval development time and pupal weight were positive at all temperatures, except at 22°C for males, although the *R* ^2^ values were small. Therefore, a longer larval period tended to result in larger pupae. Larval developmental time decreased significantly with increasing rearing temperature; whereas pupal weight was slightly but not significantly smaller at 22°C than at 25, 28 and 31°C (Figure 3, *p* > 0.05); thus, the temperature–size rule was not supported. Female pupae were significantly larger than male pupae at all temperatures (*p < *0.05).

### 3.2 Larval development time and pupal weight under field conditions

**Figure 2:**
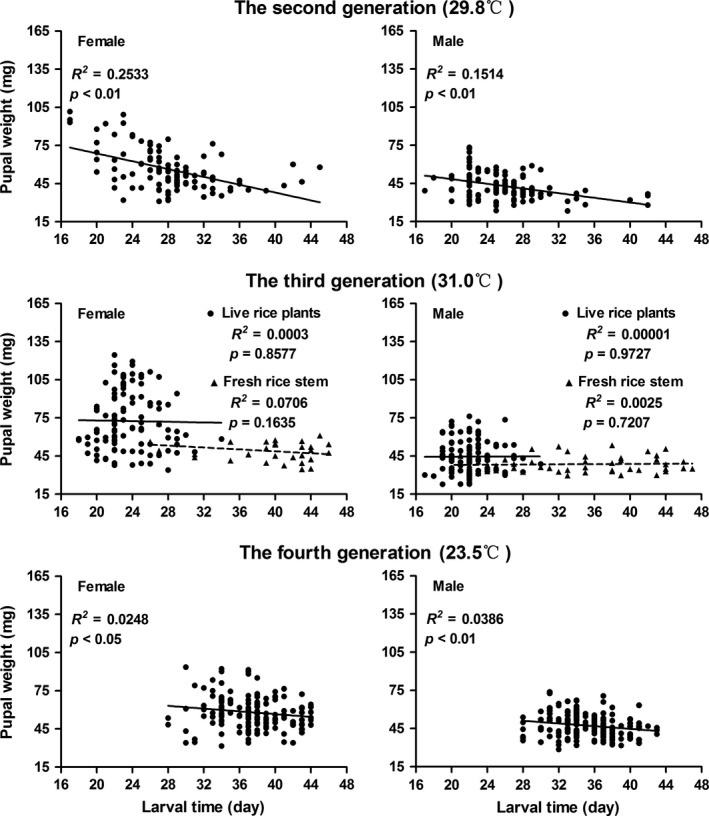
Correlation between larval development time and pupal weight of the rice stem borer *Chilo suppressalis* at the mean daily temperatures of 29.8°C (the second generation), 31°C (the third generation) and 23.5°C (the fourth generation). The data of the mean daily temperature experienced by larvae for each generation were collected from the weather station of Jiangxi Agricultural University

As shown in Figure 2, larval development time and pupal weight were negatively correlated in the second and the fourth generations (*p < *0.05) when larvae experienced a mean daily temperature of 29.8 and 23.5°C, respectively. Therefore, a longer larval period tended to result in smaller pupae. There was no correlation between development time and pupal weight in the third generation when larvae experienced a mean daily temperature of 31°C, regardless of the feeding method.

Larval development time significantly decreased as the mean daily temperature increased in both sexes (Figure 4, *p < *0.05). However, pupal weight of females was positively correlated with temperature. The highest pupal weight (72.5 mg) was at the mean daily temperature of 31°C, significantly higher than at 29.8°C (56.19 mg) and 23.5°C (52.68 mg; Figure 4, *p < *0.05), which is an example of the reverse temperature–size rule.

### 3.3 Larval development time and pupal weight when larvae fed on live rice plants or fresh rice stems

Larval development time and pupal weight were significantly affected by feeding method in both sexes when larvae were reared at the mean daily temperature of 31°C (Figure 5, *p < *0.05). Individuals reared on live rice plants showed significantly shortened larval development times (23.62 days for females, 22.10 days for males) and significantly increased pupal weights (72.5 mg for females, 44.52 mg for males) compared with those individuals fed fresh rice stems (39.21 days for females, 32.7 days for males; 49.01 mg for females, 38.51 mg for males, respectively; *p < *0.05).

Importantly, the difference in larval development time among individuals at constant temperatures was high (32–46 days) and also under field conditions of naturally changing temperatures (13–28 days).

## 4 DISCUSSION

The most interesting finding in this study is the difference between constant temperatures in the lab and field conditions. The correlations between larval development time and pupal weight were positive at constant temperatures (Figure 1), whereas the correlations were more complicated at naturally changing temperatures (Figure 2). There was no correlation between development time and pupal weight at the mean daily temperature of 31°C, whereas the correlations between the two traits were negative at the mean daily temperatures of 23.5 and 29.8°C. The results suggest that growth rates are more or less maximized in the lab, leading to the trade‐off between short development times and high pupal weights expected in the earlier literature, whereas in the field individuals react to cues from the environment and vary growth rates adaptively. Our results are consistent with the statement that natural populations of many organisms likely encounter simultaneous selection favoring different antagonistic or synergistic combinations of development time and body size (Davidowitz, Roff, & Nijhout, 2016). Therefore, the correlation between development time and body size can be positive, zero or negative (Nijhout et al., 2010).

**Figure 3:**
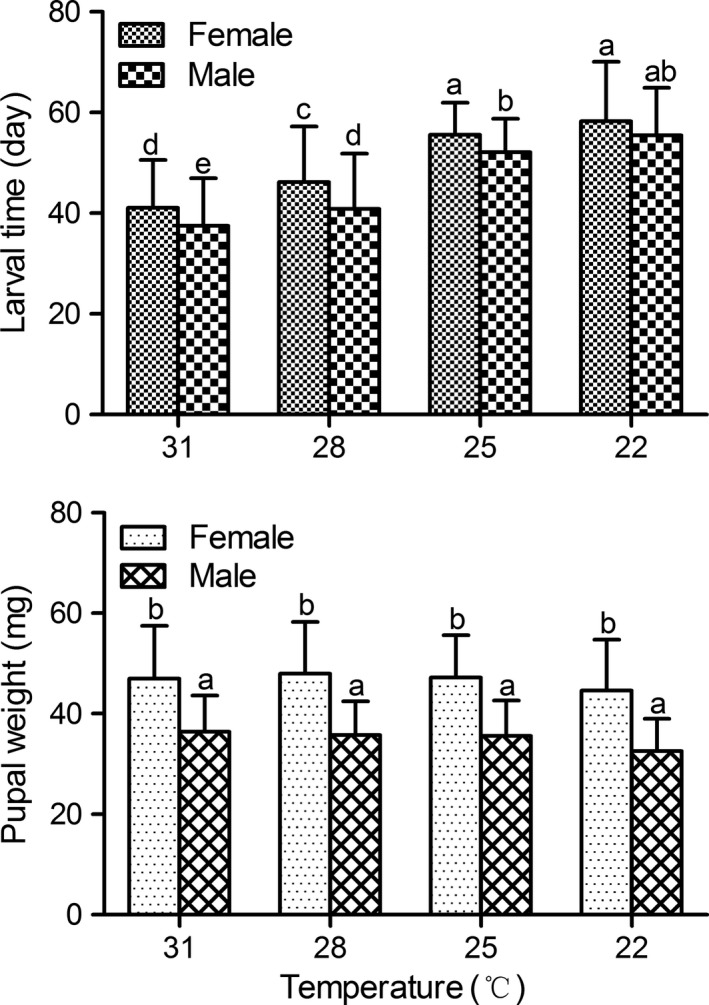
Larval development time and pupal weight of *Chilo suppressalis* at constant temperatures. Error bars indicate *SE*. Values with different lowercase letters are significantly different at the 0.05 level (*n = *46–115 for each treatment)

**Figure 4:**
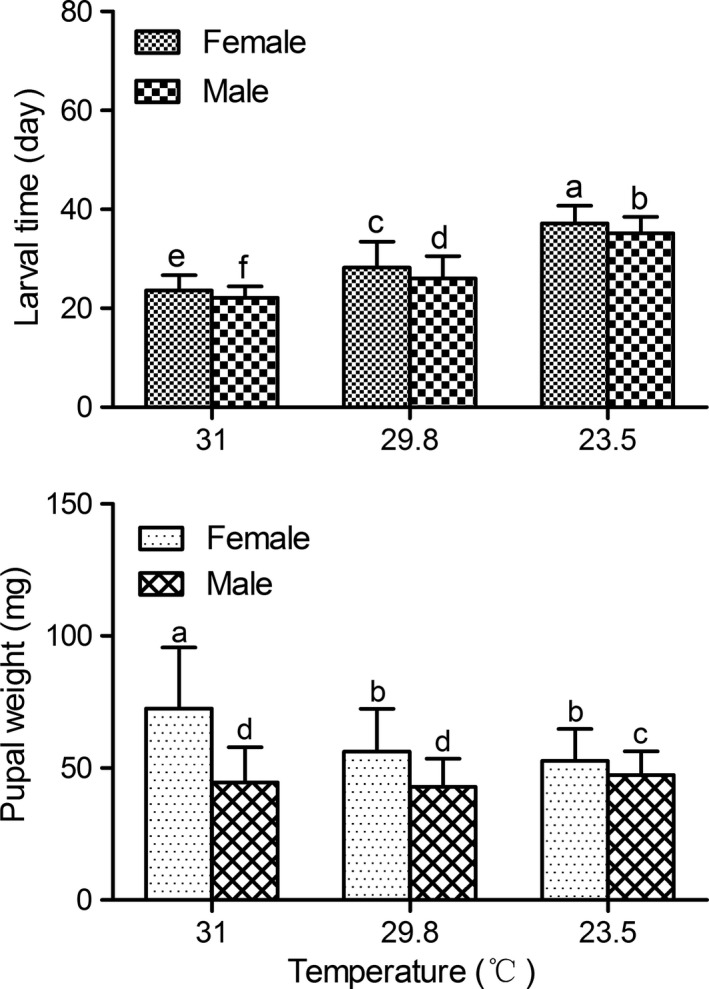
Larval development time and pupal weight of *Chilo suppressalis* under field conditions with naturally changing temperatures. Error bars indicate *SE*. Values with different lowercase letters are significantly different at the 0.05 level (*n = *107–173 for each treatment)

Most notably, in all experiments, the temperature–size rule did not apply to this moth. The pupal weight was not significantly different among the different constant temperatures, and pupae were relatively larger at the higher temperatures (Figure 3). Under field conditions, pupal weight for females was positively correlated with temperature (Figure 4), showing the reverse temperature–size rule. Therefore, development time and body weight in this moth were not strongly correlated with one another, and larger pupae could be also attained after a short development time. Thus, fast‐developing individuals also grew larger than less vigorous ones with longer development periods and resulting smaller pupae.

Analyses of life history strategies are usually based on two assumptions: selection maximizes the fitness of an animal, and a trade‐off constrains possible life history combinations (Roff, 1992; Stearns, 1992). In the moth *C. suppressalis,* we did not detect a trade‐off between development time and body weight under field conditions. On the contrary, the highest mean daily temperature of 31°C resulted in the shortest larval development time of females (23.62 ± 3.09 days) and the largest pupal weight (72.50 ± 23.21 mg), compared with the mean temperatures of 29.8°C (28.22 ± 5.30 days for larval development time, 56.19 ± 16.19 mg for pupal weight) and 23.5°C (37.18 ± 3.58 days for larval development time, 52.68 ± 12.08 mg for pupal weight). The shorter development time caused by high temperature that resulted in significantly larger body weight was an indication that increasing temperature had a stronger effect on growth rate than on development rate (Van Der Have & De Jong, 1996), i.e., the biomass accumulation rate was more temperature sensitive than the development rate during the larval development period (Angilletta & Dunham, 2003; Zuo, Moses, West, Hou, & Brown, 2012). Such a life history strategy reflects an evolutionary adaptation. According to six years of field observations on the incidence of winter diapause of *C. suppressalis*, most or almost all individuals that hatched after mid‐August enter diapause in response to gradually shortened autumnal daylenght, despite the high temperatures that prevail during August (the mean daily temperature was approximately 28°C; Xiao et al., 2010). Therefore, because high temperature resulted in high body weight, this subtropical population can attain a higher larval body weight at maturity before hibernation. Larger larvae would be expected to have higher survival rates during winter and greater performance after hibernation (ChownScholtz, Klok, Joubert, & Coles, 1995; Davidowitz, 2008; Peters, 1986). The same, those nondiapause individuals produced under high temperatures that prevail during August were also larger with higher fecundity (unpublished data) and produced overwintering generation in early September. Thus the moth has the opportunity to build up a huge overwintering population. This is the reason why larvae from the first generation always cause great damage to the early rice (Xiao et al., 2010).

The large pupal weight attained with a short larval period under high temperature in *C. suppressalis* implies that global climate warming may have beneficial effects on the fecundity of this moth, making this pest an even greater threat. Similar results were also found in a temperate population of *C. suppressalis* (Fu et al., 2016) and in the Asian corn borer *O. furnacalis* (Xiao et al., 2016).

**Figure 5:**
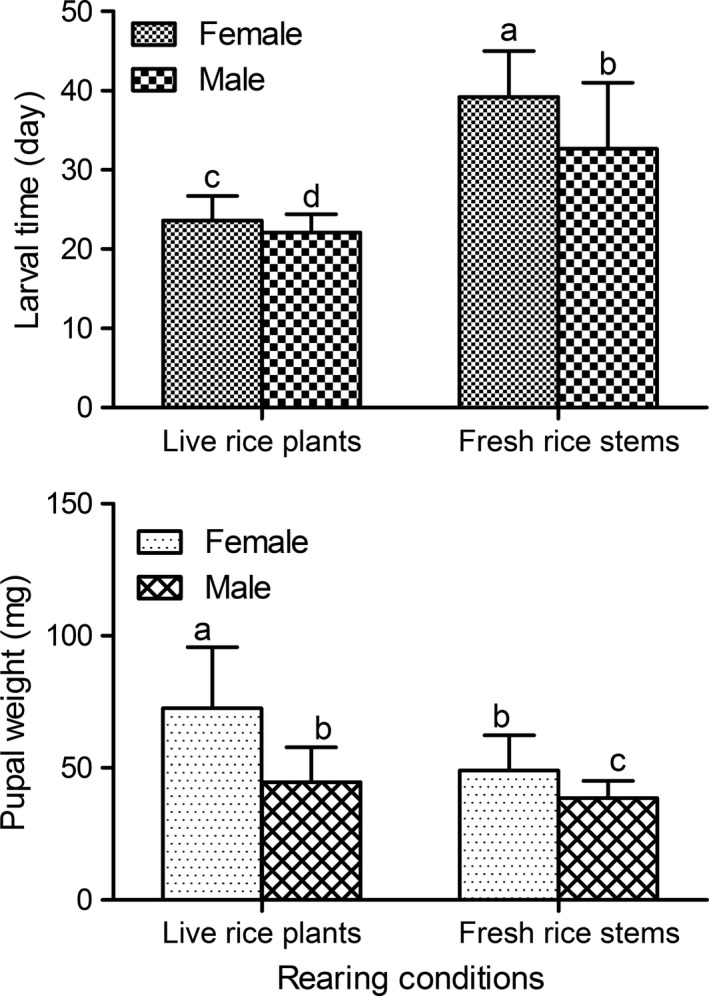
Comparison of larval development time and pupal weight of *Chilo suppressalis* under the mean daily temperature of 31°C when larvae that hatched the same day were reared on live rice plants or on fresh rice stems. Error bars indicate *SE*. Values with different lowercase letters are significantly different at the 0.05 level (*n = *29–108 for each treatment)

Influence of nutrient quantity and quality on development and body size has been reported in several insects (Blanckenhorn, 1998; Davidowitz, D’Amico, & Nijhout, 2004; Gebhardt & Stearns, 1988; Ikeya, Galic, Belawat, Nairz, & Hafen, 2002; Kause et al., 2001; Uhl et al., 2004). In a previous study with this rice borer, larvae reared with rice plants showed significantly longer larval development time and attained significantly smaller pupae than those fed with water‐oats (Xiao, Xue, Liu, & Zhu, 2005). Notably, in the present study, the feeding method significantly affected larval development time and pupal weight (Figure 5). Larval development time of individuals fed the live rice plants (23.62 ± 3.09 days for females, 22.10 ± 2.31 days for males) was significantly shorter than the development time of those fed the fresh rice stems (39.21 ± 5.83 for females, 32.70 ± 8.31 days for males). Additionally, pupae of larvae fed the live rice plants (72.50 ± 23.21 mg for females, 44.52 ± 13.28 mg for males) were significantly larger than pupae of those fed the fresh rice stems (49.01 ± 8.26 mg for females, 38.51 ± 6.49 for males). The differences in the two traits resulting from the two feeding methods might be explained because the fresh stems were not viable; whereas live rice plants could transport nutrients. Thus, to better understand the evolution of life history traits in this rice stem borer, a field experiment is required. In *Drosophila*, nutrients affect growth rate and body size primarily by altering the secretion of insulin‐like peptides, and genetic defects in components of the insulin‐signaling pathway result in long development times and small flies (Ikeya et al., 2002). Therefore, we assumed that similar mechanisms might be involved in larvae fed fresh rice stems compared with those fed the live rice plants.

## 5 CONCLUSIONS

The observations on larval development time and pupal weight under laboratory and field conditions in *C. suppressalis* revealed that the relationships between the two traits could be positive, zero or negative, depending on the rearing conditions. Because high temperature resulted in both large pupae and short larval development time in *C. suppressalis*, a trade‐off was not apparent between the development time and body weight under field conditions. Such a reverse temperature‐body size rule may indicate a strategy to ensure overwintering larvae acquire a high body weight before hibernation. Thus, the inference is that global climate warming during autumn in subtropical regions will likely make this pest a more severe problem. Our finding that feeding method significantly affected larval development time and pupal weight emphasizes the importance of conducting field experiments in the study of life history traits.

## 6 DATA ACCESSIBILITY STATEMENT

We agree to deposit our data to a public repository.

## CONFLICT OF INTEREST

None declared.

## AUTHOR CONTRIBUTIONS

Fangsen Xue conceived and designed research. Xiao‐Long Huang and Hai‐Min He conducted experiments and analyzed data. Fangsen Xue and Lan Xiao wrote the manuscript. All authors read and approved the manuscript.
